# Detection of Degraded Star Observation Using Singular Values for Improved Attitude Determination

**DOI:** 10.3390/s24020593

**Published:** 2024-01-17

**Authors:** Kiduck Kim

**Affiliations:** Korea Aerospace Research Institute, Daejeon 34133, Republic of Korea; kdkim330@naver.com; Tel.: +82-10-3718-5262

**Keywords:** star, star tracker, vector observation, singular value, attitude determination

## Abstract

This study introduces an innovative approach aimed at enhancing the accuracy of attitude determination through the computation of star observation quality. The proposed algorithm stems from the inherent invariance of singular values under attitude transformations, leveraging the concept of assessing error magnitude through the deviation of singular values. Quantization becomes imperative to employ this error magnitude as a weighting factor within the attitude determination process. To fulfill this purpose, this study applies p-value hypothesis testing to calculate quantized error levels. Simulation results validate that the calculated weights derived from the proposed algorithm lead to a discernible enhancement in attitude determination performance.

## 1. Introduction

Accurate attitude determination is an essential requirement for the success of a spacecraft in achieving the specified mission objectives [1]. Since even a minor deviation in the spacecraft’s attitude can lead to significant consequences, more precise attitude determination is being emphasized to mitigate the risk of potential bottlenecks that could limit the success of space missions. Among the various attitude determination sensors available to satisfy this requirement, star trackers stand out as key devices that provide the highest level of accuracy in on-orbit attitude determination [2,3].

After the star is projected onto the image plane of the star tracker, it goes through a series of workflows: centroid extraction, star identification from a star catalog, and attitude determination [4]. For precise attitude determination, the extracted center position of the star from the image must be accurate [5]. However, with the recent advancements in maneuvering performance, many satellites have gained the potential for agile maneuvers [6]. These maneuvers cause image smearing during the exposure time of the star tracker [7], leading to a decrease in the accuracy of the centroid error [8]. This effect is heightened by increased maneuvering speeds [9]. Such a phenomenon is particularly noticeable in dim stars with lower signal-to-noise ratios when compared to bright stars [10]. Several studies have been proposed to attain comparable performance in such situations [11,12,13,14]. However, there inevitably exists a degradation in the accuracy of the centroid error, with the magnitude of this degradation varying from star to star based on their respective signal-to-noise ratios [15].

The signal-to-noise ratio is related to the brightness of the star. Nevertheless, owing to the ambiguity in magnitude information [16,17], it should not be depended upon to determine the extent of the error. Alongside signal-to-noise ratio, noise characteristics such as distortion, image degradation due to temperature, and starlight deflection can also introduce varying degrees of noise among different stars. Until now, several studies have presented strategies for utilizing star sensors in situations involving degradation. However, the predominant focus has been on strengthening the robust detection [18] and identification [8,19] of stars. As a result, the need arises for a method to quantify the varying levels of accuracy among stars and to incorporate this information into attitude determination for enhanced precision.

Accurate attitude determination also necessitates a robust star identification process. The occurrence of false matches during identification is another factor that diminishes the accuracy and reliability of attitude determination. While research in star identification has made significant strides, recent advancements have introduced algorithms that employ pattern-based feature extraction methods [20]. It is widely acknowledged that pattern-based recognition algorithms perform optimally when a substantial number of stars are present within an image frame, ensuring the distinctiveness of each pattern [21,22]. While a larger quantity stars can indeed enhance the stability of matching performance, the inclusion of dim stars can intensify the centroid error due to their inherently low signal-to-noise ratios. Hence, it becomes crucial to achieve not only precise centroid error but also robust star identification to ensure accurate attitude determination. Nonetheless, these two prerequisites present conflicting challenges, which unavoidably result in variations in accuracy among individual stars.

Therefore, this study proposes an innovative approach to assess the quality of star observations preceding the attitude determination, with the aim of enhancing accuracy. The primary contribution lies in the computation of individual star observation quality by utilizing the singular values among stars. Singular values have also been adopted in star identification techniques [23,24] and calibration [25] due to their insensitivity to attitude changes, making them suitable as reference values for determining the degree of error in star observations. To quantify this, we introduce p-value hypothesis testing to derive a quantized error level. In the end, this value can serve as a weighting factor in the attitude determination process, thereby contributing to the improvement of accuracy.

The organization of this paper can be summarized as follows. Section 2 furnishes the requisite background information relevant to this study. Moving ahead, Section 3 outlines the procedure for computing the quality level based on singular values, which constitutes the fundamental essence of this research. In Section 4, we validate the accuracy of the proposed method in quantifying the degree of error through simulation results and further analyze its practicality by applying it to attitude determinations. Lastly, Section 5 concludes by summarizing the main findings of the research presented in this paper.

## 2. Background

### 2.1. Invariance of Singular Values

In this section, our objective is to offer concise insights to readers who might not be acquainted with the attitude-invariant properties of singular values and attitude determination. Singular value decomposition techniques [26] have found extensive application in domains like signal and image processing [27], as well as attitude estimation [28], enabling the extraction of valuable insights from correlation matrices. In fact, this methodology is renowned as one of the most resilient algorithms in the realm of numerical algebra [29].

**Figure 1:**
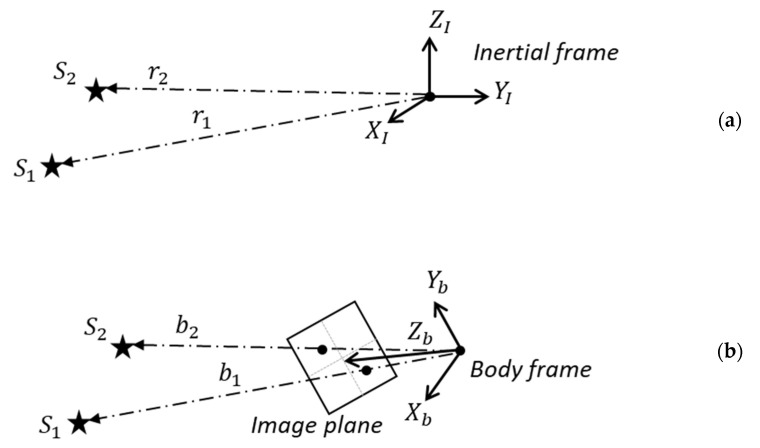
A set of two stars seen from two different reference frames: (**a**) an inertial reference frame; (**b**) the body reference frame.

A vector observation in a star tracker represents the relationship between the unit vector $r_{i}$ of the $i_{th}$ star, presented in the inertial frame, and the unit vector $b_{i}$ presented in the body frame, as illustrated in Figure 1. This relationship can be mathematically expressed through a matrix operation that rotates an arbitrary vector from the inertial frame to the body frame, which is defined as

(1) $$b_{i}=C_{I}^{B}\cdot r_{i}$$

where $C_{I}^{B}$ represents the directional cosine matrix (DCM) containing the attitude information in the body frame relative to the inertial frame. The subscript ‘I’ and the superscript ‘B’ signify the inertial and body frames, respectively.

Notably, this matrix is characterized by its property of being a unitary matrix that conforms to the following expression:

(2) $$CC^{T}=C^{T}C=I_{3\times 3}$$

(3) $$C^{-1}=C^{T}$$

where $I_{3\times 3}$ represents an identity matrix of size 3 × 3. If N stars are observed from the star tracker, these operations can be extended using $C_{I}^{B}$. The vector matrices for N stars observed in the inertial frame and the body frame are denoted as $R_{3\times N}$ and $B_{3\times N}$, respectively, with subscripts indicating that the matrices have sizes of 3 × *N*.

(4) $$B_{3\times N}=C_{I}^{B}\cdot R_{3\times N}$$

The observation vector matrices $R_{3\times N}$ and $B_{3\times N}$ are defined as follows:

(5) $$R_{3\times N}=\left[\begin{array}{cc} \begin{array}{cc} r_{1}, & r_{2}, \end{array} & \begin{array}{cc} \cdots & r_{N} \end{array} \end{array}\right]$$

(6) $$B_{3\times N}=\left[\begin{array}{cc} \begin{array}{cc} b_{1}, & b_{2}, \end{array} & \begin{array}{cc} \cdots & b_{N} \end{array} \end{array}\right]$$

These $R_{3\times N}$ and $B_{3\times N}$ matrices can be factorized through singular value decomposition.

(7) $$R_{3\times N}=P_{r}D_{r}Q_{r}^{T}=\sum_{i=1}^{3}p_{r_{i}}\sigma_{r_{i}}q_{r_{i}}^{T}$$

(8) $$B_{3\times N}=P_{b}D_{b}Q_{b}^{T}=\sum_{i=1}^{3}p_{b_{i}}\sigma_{b_{i}}q_{b_{i}}^{T}$$

where $P_{r}$ and $P_{b}$ are 3 × 3 orthogonal matrices, representing the normalized eigenvectors of $R_{3\times N}{R_{3\times N}}^{T}$ and $B_{3\times N}{B_{3\times N}}^{T}$, respectively. $D_{r}$ and $D_{b}$ are 3 × *N* diagonal matrices, with their components being the singular values $\sigma_{r_{i}}$ and $\sigma_{b_{i}}$ of $R_{3\times N}$ and $B_{3\times N}$. $Q_{r}$ and $Q_{b}$ are N×N orthogonal matrices, denoting the normalized eigenvectors of ${R_{3\times N}}^{T}R_{3\times N}$ and ${B_{3\times N}}^{T}B_{3\times N}$, respectively. Since a star’s unit vector signifies its orientation in three-dimensional space, if more than three stars are observed (N≥3), both matrices $R_{3\times N}$ and $B_{3\times N}$ will have a rank of 3. Therefore, when observing more than three stars, the computation of three non-zero singular values will always be feasible.

By multiplying Equation (4) with the transpose of $B_{3\times N}$, we can reformulate it as Equation (9). This transformation exhibits the characteristics of a similarity transformation, a consequence of the unitary property of the directional cosine matrix $C_{I}^{B}$.

(9) $$B_{3\times N}B_{3\times N}^{T}=C_{I}^{B}\cdot R_{3\times N}R_{3\times N}^{T}\cdot {C_{I}^{B}}^{-1}$$

The expression in Equation (9) depicts a similarity transformation between two symmetric matrices, namely $R_{3\times N}{R_{3\times N}}^{T}$ and $B_{3\times N}{B_{3\times N}}^{T}$, both having a size of 3 × 3. Upon substituting Equations (7) and (8) into both sides of Equation (9), the following outcome emerges:

(10) $$C_{I}^{B}\cdot R_{3\times N}R_{3\times N}^{T}\cdot {C_{I}^{B}}^{-1}=C_{I}^{B}P_{r}D_{r}Q_{r}^{T}Q_{r}D_{r}^{T}P_{r}^{T}{C_{I}^{B}}^{-1}=C_{I}^{B}P_{r}{\tilde{S}}_{r}P_{r}^{T}{C_{I}^{B}}^{-1}$$

(11) $$B_{3\times N}B_{3\times N}^{T}=P_{b}D_{b}Q_{b}^{T}Q_{b}D_{b}^{T}P_{b}^{T}=P_{b}{\tilde{S}}_{b}P_{b}^{T}$$

Equation (12) can be derived substituting Equations (10) and (11) into Equation (9), as given below:

(12) $$P_{b}{\tilde{S}}_{b}P_{b}^{T}=C_{I}^{B}P_{r}{\tilde{S}}_{r}P_{r}^{T}{C_{I}^{B}}^{-1}$$

Here, ${\tilde{S}}_{r}$ and ${\tilde{S}}_{b}$ represent 3 × 3 diagonal matrices with the squares of the three eigenvalues, $\sigma_{r_{i}}^{2}$ and $\sigma_{b_{i}}^{2}$ (with i=1,2,3), as their respective components.

(13) $${\tilde{S}}_{r}=D_{r}D_{r}^{T}$$

(14) $${\tilde{S}}_{b}=D_{b}D_{b}^{T}$$

The relationship between the eigenvalues $\sigma_{r_{i}}$ and $\sigma_{b_{i}}$ is conserved throughout the similarity transformation in Equation (9), allowing us to deduce

(15) $${\tilde{S}}_{r}={\tilde{S}}_{b}$$

or

(16) $$\begin{array}{ccc} \sigma_{r_{1}}^{2}=\sigma_{b_{1}}^{2}, & \sigma_{r_{2}}^{2}=\sigma_{b_{2}}^{2}, & \sigma_{r_{3}}^{2}=\sigma_{b_{3}}^{2} \end{array}$$

This suggests that the singular values of $R_{3\times N}$ and $B_{3\times N}$ remain unaltered, regardless of any shifts in coordinates resulting from attitude transformations.

(17) $$\begin{array}{ccc} \sigma_{r_{1}}=\sigma_{b_{1}}, & \sigma_{r_{2}}=\sigma_{b_{2}}, & \sigma_{r_{3}}=\sigma_{b_{3}} \end{array}$$

### 2.2. Attitude Determination

Attitude can be determined using N known nonlinear vectors, which are obtained from the vectors in the inertial frame along with their corresponding observations in the body frame [30]. This indicates the N star observation vector matrices $R_{3\times N}$ and $B_{3\times N}$ mentioned earlier. Attitude determination plays a crucial role in satellite control and adopts an algebraic approach for reconstructing attitude. The primary objective of attitude determination algorithms is to minimize a loss function, known as Wahba’s problem, as illustrated in the equation below [31].

(18) $$I\left(C_{I}^{B}\right)=\frac{1}{2}\sum_{i=1}^{N}k_{i}{\left\|b_{i}-C_{I}^{B}r_{i}\right\|}^{2}$$

Here, $k_{i}$ represents the confidence level of the $i_{th}$ observation with a non-negative weight. To date, one of the most widely adopted methods for addressing Wahba’s problem is the QUEST (QUaternion ESTimator) algorithm [32]. Originating from the Davenport q-method [33], which offered an initial solution to Wahba’s problem, the QUEST algorithm has the capacity to identify the optimal solution for Wahba’s problem when presented with two or more vector observations.

The loss function enables us to formulate the subsequent gain function. As such, it becomes a problem of finding the optimal solution that maximizes this gain function.

(19) $$g\left(C_{I}^{B}\right)=1-I\left(C_{I}^{B}\right)=\sum_{i=1}^{N}k_{i}b_{i}^{T}C_{I}^{B}r_{i}$$

The gain function is derived through the following trace operation:

(20) $$g\left(C_{I}^{B}\right)=tr\left[C_{I}^{B}M^{T}\right]$$

Here, ‘tr’ represents a trace operation. The matrix M and the normalized weight $w_{i}$ are defined as

(21) $$M=\sum_{i=1}^{N}w_{i}b_{i}r_{i}^{T}$$

(22) $$w_{i}=\frac{k_{i}}{\sum_{i=1}^{N}k_{i}}$$

The attitude $C_{I}^{B}$ can be represented using the corresponding quaternion $Q={\left[\begin{array}{cc} q_{0}, & q^{T} \end{array}\right]}^{T}$ as follows. Here, ${\left[q\right]}_{\times }$ refers to skew-symmetric matrices with the vector part of the quaternion Q.

(23) $$C_{I}^{B}\left(Q\right)=\left(q_{0}^{2}-{\left\|q\right\|}^{2}\right)I_{3\times 3}+2qq^{T}+2q_{0}{\left[q\right]}_{\times }$$

By substituting the above expression into the previous one, the gain function is summarized in the subsequent bilinear form:

(24) $$g\left(C_{I}^{B}\left(Q\right)\right)=Q^{T}ΚQ$$

where K is the following matrix with a size of 4 × 4.

(25) $$K=\left[\begin{array}{cc} tr\left\{M\right\} & {\left(\sum_{i=1}^{N}w_{i}b_{i}\times r_{i}\right)}^{T}\\ \sum_{i=1}^{N}w_{i}b_{i}\times r_{i} & M+M^{T}-tr\left\{M\right\}I_{3\times 3} \end{array}\right]$$

The optimal quaternion $Q_{opt}$, obtained via the QUEST algorithm, corresponds to the eigenvector coupled with the largest eigenvalue of the matrix K.

(26) $$ΚQ_{opt}=\lambda_{max}Q_{opt}$$

In terms of $w_{i}$, it is important to note that existing works solely mention the constraint that the set of weights sums to one, without any loss of generality. Therefore, we embark on our approach by introducing the concept of enhancing attitude accuracy by reflecting the individual confidence of the observations into the weights $w_{i}$.

In summary, the invariance of singular values to attitude variations can serve as a reference value. The proposed work suggests an innovative approach by computing the divergence between the singular value derived from observed stars and employing it as a weighting factor in the attitude determination process, thereby enhancing accuracy. This novel aspect stands as the primary contribution of the proposed work, and the following section provides a detailed explanation of this approach.

## 3. Quality Level Calculation

### 3.1. Variation of Singular Values

Before proceeding with the calculation of the quality value, we examined whether singular values indeed remain unaltered with respect to attitude variations and assessed the magnitude of changes induced in singular values when there is image noise. For the simulations, synthesized star images are generated using the well-known Hipparcos catalog as reference star catalog [34]. For each simulation, random attitude is provided to emulate the star tracker’s boresight direction to view various directions within the celestial sphere.

**Figure 2:**
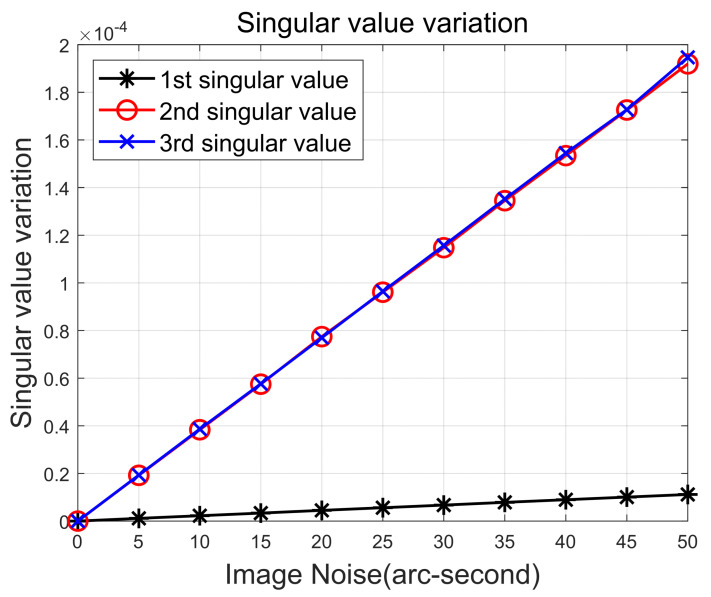
Variation of singular values at each noise level.

The field of view, pixel size, and resolution of the star tracker are assumed to be 12 degrees, $10^{-5}$ m, and 512 by 512, respectively. We introduced an error in the center position of the star projected onto the image plane and varied its magnitude to examine the effects on the singular values. The introduced error follows a random Gaussian noise distribution, and Figure 2 displays the average change after performing 10,000 simulations for each error size.

The three calculated singular values are ordered, with the first singular value defined as the one with the largest magnitude, followed by the remaining singular values in descending order of their magnitudes. It is noticeable that the change in singular value tends to increase linearly with the magnitude of the noise. This result suggests that quality can be evaluated by analyzing the error size introduced into the observations based on the differences in singular values.

Moreover, it is noticeable that the alteration in the first singular value is significantly smaller compared to the other two singular values. It is crucial to understand the covariance of singular value changes under normal sensor conditions, as it is a key factor in calculating the quality value, which will be discussed in a later section. In this study, the error under normal star tracker conditions is assumed to be 10 arc-seconds, and the changes and standard deviations of each singular value at that time are summarized in Table 1.

### 3.2. Quality Value Calculation

The remaining question is how to quantify the quality of each observation. In star trackers, we can attribute noise to centroid errors resulting from satellite maneuvers, temperature fluctuations, distortion, and more. Bias can be linked to star deflection and false matches in the identification algorithm. This study suggests utilizing the singular value to quantify both error and bias, leveraging its inherent invariance to attitude changes in an ideal scenario.

As outlined in the background, a minimum of three stars is necessary to calculate the singular value. The subsequent question is to identify which stars, employed in this calculation, might exhibit error and bias. To address this, we specifically choose three stars, the minimum requisite for calculating the singular value, and create a subset as follows:

(27) $$S_{Sub_{j}}=\left\{\begin{array}{ccc} s_{l}, & s_{m}, & s_{n} \end{array}\right\}\subset U$$

where U denotes the set comprising all star observations:

(28) $$U=\left\{\begin{array}{cc} \begin{array}{cc} s_{1}, & s_{2}, \end{array} & \begin{array}{cc} \cdots & s_{N} \end{array} \end{array}\right\}$$

The singular value computed for a specific subset can be represented in the following manner:

(29) $${\left[\begin{array}{c} {{SV}_{1}}_{{sub}_{j}}\\ {{SV}_{2}}_{{sub}_{j}}\\ {{SV}_{3}}_{{sub}_{j}} \end{array}\right]}_{meas}={\left[\begin{array}{c} {{SV}_{1}}_{{sub}_{j}}\\ {{SV}_{2}}_{{sub}_{j}}\\ {{SV}_{3}}_{{sub}_{j}} \end{array}\right]}_{true}+{\left[\begin{array}{c} v_{{SV}_{1}}\\ v_{{SV}_{2}}\\ v_{{SV}_{3}} \end{array}\right]}_{{sub}_{j}}+{\left[\begin{array}{c} b_{{SV}_{1}}\\ b_{{SV}_{2}}\\ b_{{SV}_{3}} \end{array}\right]}_{{sub}_{j}}$$

with $v_{{SV}_{i}}$ and $b_{{SV}_{i}}$ signifying the noise and bias associated with the $i_{th}$ singular value calculated from the subset ${sub}_{j}$. Calculating the true value of the singular value is achievable using database information for a recognized star. When we rewrite the preceding expression as depicted below, the difference between the observed value and the true singular value becomes expressible solely as a function of noise and bias, irrespective of their specific values.

(30) $${\left[\begin{array}{c} {{SV}_{1}}_{{sub}_{j}}\\ {{SV}_{2}}_{{sub}_{j}}\\ {{SV}_{3}}_{{sub}_{j}} \end{array}\right]}_{meas}-{\left[\begin{array}{c} {{SV}_{1}}_{{sub}_{j}}\\ {{SV}_{2}}_{{sub}_{j}}\\ {{SV}_{3}}_{{sub}_{j}} \end{array}\right]}_{true}={\left[\begin{array}{c} v_{{SV}_{1}}\\ v_{{SV}_{2}}\\ v_{{SV}_{3}} \end{array}\right]}_{{sub}_{j}}+{\left[\begin{array}{c} b_{{SV}_{1}}\\ b_{{SV}_{2}}\\ b_{{SV}_{3}} \end{array}\right]}_{{sub}_{j}}$$

Therefore, the procedure for evaluating the error level can be characterized as a problem of quantifying the extent of bias within the singular value difference ${\left({SV}_{diff}\right)}_{{sub}_{j}}$ of the subgroup ${sub}_{j}$, as defined below.

(31) $${\left({SV}_{diff}\right)}_{{sub}_{j}}={\left[\begin{array}{c} {{SV}_{1}}_{{sub}_{j}}\\ {{SV}_{2}}_{{sub}_{j}}\\ {{SV}_{3}}_{{sub}_{j}} \end{array}\right]}_{meas}-{\left[\begin{array}{c} {{SV}_{1}}_{{sub}_{j}}\\ {{SV}_{2}}_{{sub}_{j}}\\ {{SV}_{3}}_{{sub}_{j}} \end{array}\right]}_{true}$$

To address this issue, we establish the null hypothesis $H_{0}$ and the alternative hypothesis $H_{1}$ and introduce statistical hypothesis testing. Hypothesis testing is a significance test that determines whether to accept or reject the null hypothesis. $H_{0}$ represents the ideal scenario where the singular value derived from $H_{0}$ is devoid of errors and biases.

(32) $$H_{0}={\left(VB\right)}_{{sub}_{j}}=0$$

(33) $$H_{1}={\left(VB\right)}_{{sub}_{j}}\neq 0$$

where

(34) $${\left(VB\right)}_{{sub}_{j}}={\left[\begin{array}{c} v_{{SV}_{1}}\\ v_{{SV}_{2}}\\ v_{{SV}_{3}} \end{array}\right]}_{{sub}_{j}}+{\left[\begin{array}{c} b_{{SV}_{1}}\\ b_{{SV}_{2}}\\ b_{{SV}_{3}} \end{array}\right]}_{{sub}_{j}}$$

By utilizing the error covariance value in the normal situation of the star tracker, ${\left({SV}_{diff}\right)}_{{sub}_{j}}$ is normalized as follows:

(35) $$\Gamma \left({\left({SV}_{diff}\right)}_{{sub}_{j}}\right)={\left({\left({SV}_{diff}\right)}_{{sub}_{j}}\right)}^{T}\cdot {R_{sv}}^{-1}\cdot {\left({SV}_{diff}\right)}_{{sub}_{j}}$$

Since $\Gamma \left({\left({SV}_{diff}\right)}_{{sub}_{j}}\right)$ represents the dot product of identical vectors with consideration for variance, it follows a chi-square distribution, and $R_{sv}$ is the error covariance of the singular value in the normal situation.

(36) $$R_{sv}=diag\left({\left[\begin{array}{ccc} \sigma_{sv1}^{2}, & \sigma_{sv2}^{2}, & \sigma_{sv3}^{2} \end{array}\right]}^{T}\right)$$

The *p*-value represents the probability of observing an event of a particular magnitude under the assumption that the null hypothesis is true. It is defined by $P_{{sub}_{j}}$ as follows:

(37) $$P_{{sub}_{j}}=P\left({\left({SV}_{diff}\right)}_{{sub}_{j}}\right)=F_{0}\left[Χ\geq \Gamma \left({\left({SV}_{diff}\right)}_{{sub}_{j}}\right)\right]$$

$F_{0}$ stands for the cumulative probability function of the null hypothesis. When the $\Gamma \left({\left({SV}_{diff}\right)}_{{sub}_{j}}\right)$ value is equal to 0, it completely matches the null hypothesis, resulting in a *p*-value of 1. Therefore, the *p*-value, denoted as $P_{{sub}_{j}}$, indicates the likelihood of how closely the subgroup ${sub}_{j}$ belongs to $H_{0}$ and ranges between 0 and 1. A higher $P_{{sub}_{j}}$ implies stronger support for $H_{0}$, indicating higher quality for the observations in ${sub}_{j}$. Consequently, the individual quality value $q_{s_{i}}$ for the $i_{th}$ star can be computed by aggregating the p-values from all subgroups containing that star.

(38) $$q_{s_{i}}=\frac{1}{n_{sub_{j}}}\sum_{s_{i}\;\in \;sub_{j}}P_{{sub}_{j}}$$

(39) $$n_{sub_{j}}=\frac{\left(N-1\right)\left(N-2\right)}{2}$$

where $n_{{sub}_{j}}$ represents the number of all subsets containing the $i_{th}$ star $s_{i}$ and N is the total number of observed stars. The calculated $q_{s_{i}}$ has the same meaning as $k_{i}$ in Equation (18). Therefore, it can be substituted into Equation (22) to calculate the normalized weight of the attitude determination process. This adjustment allows us to assign more weight to observations with higher quality, ultimately leading to improved attitude accuracy.

## 4. Simulation Results

### 4.1. Quality Value Verification

To verify the performance of the proposed approach, the initial step involves checking that the quality value calculation is carried out correctly as intended. Subsequently, the effectiveness of the calculated quality value when applied to the attitude determination process is assessed. As previously mentioned, it is assumed that the star tracker would exhibit a standard error of 10 arc-seconds (1σ) under normal operating conditions, as discussed earlier. The $R_{sv}$ value used in the quality value calculation is based on this assumption and made use of the values provided in Table 1.

Several scenarios are then considered in which larger than normal errors are intentionally added to specific stars, and Table 2 summarizes them. In all scenarios, six stars are initially recognized by star tracker. Scenario 1 involves the introduction of errors of 25 arc-seconds and 60 arc-seconds to the 2nd and 6th stars, respectively. In Scenario 2, errors of 35 arc-seconds and 70 arc-seconds are added to the 2nd and 6th stars, respectively. Quality values are calculated for 100 s in both Scenarios 1 and 2, confirming that varying degrees of error yield the correct quality values. In Scenario 3, the quality value is computed over a 200-s duration, with the error size being altered midway, and the outcomes are observed. Implemented errors in the scenario is assumed to follow a random Gaussian distribution.

To ensure a reliable acquisition of stars and facilitate the analysis of simulation results, it is assumed that the true attitude of the star tracker is maintained in a stationary state. The star tracker’s update rate is 1Hz, and the simulation time is in the time-scaled values of this rate. For instance, if 100 s of simulation time elapse, it means the acquisition of 100 image frames. Each image frame consistently captures six stars, and the identities of these stars in the catalog are summarized in Table 3.

**Figure 3:**
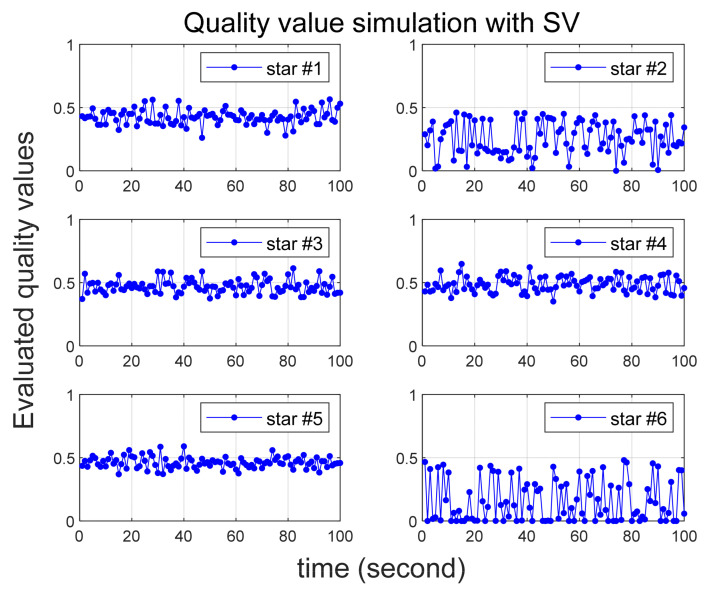
Quality value calculation results under Scenario 1.

**Figure 4:**
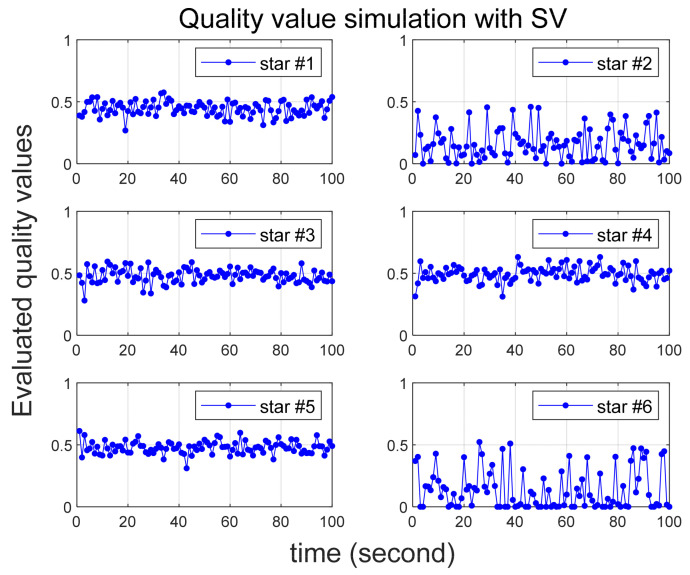
Quality value calculation results under Scenario 2.

Figure 3 and Figure 4 illustrate the simulation outcomes for Scenarios 1 and 2, respectively. These results affirm the effective performance of the proposed algorithm in calculating the quality value, which accurately reflects the magnitude of errors present in the observed stars. Notably, the quality values of the 6th star in Scenario 1 and Scenario 2, both of which contain errors exceeding 60 arc-seconds, significantly larger than the normal situation, are observed to be nearly zero. These results suggest that when incorrect star observations arise due to sensor malfunction or high rate slewing motion, the quality value computation can effectively filter out these erroneous observations before entering the attitude determination process. Furthermore, the errors introduced to the 2nd star in Scenario 1 and Scenario 2 exhibit slight variations. Upon comparing these two cases, it becomes evident that the 2nd star in Scenario 2, characterized by a larger error, possesses a relatively smaller quality value.

**Figure 5:**
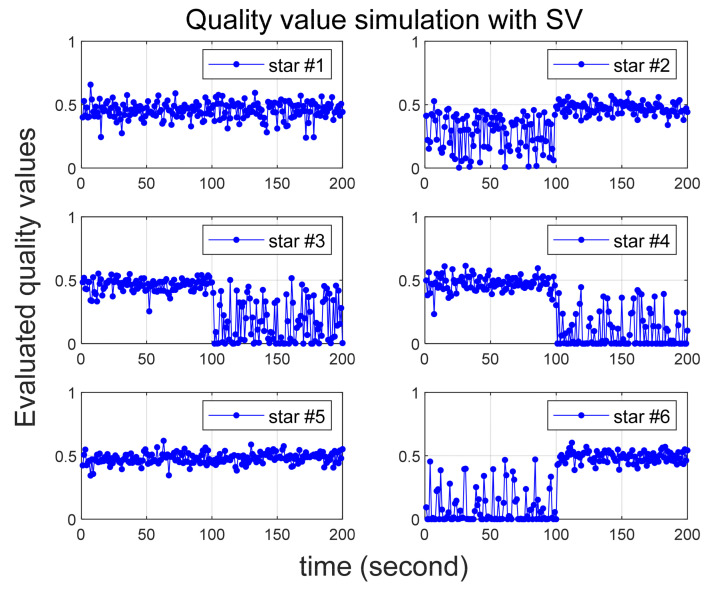
Quality value calculation results under Scenario 3.

The results of Scenario 3 are presented in Figure 5. It can be observed that the error level in the observation changes after 100 s, and the corresponding shift in quality levels is accurately reflected. From the simulation results, it can be inferred that the proposed approach is capable of effectively accommodating the existing errors in the observations and can serve as a valuable weighting factor.

**Figure 6:**
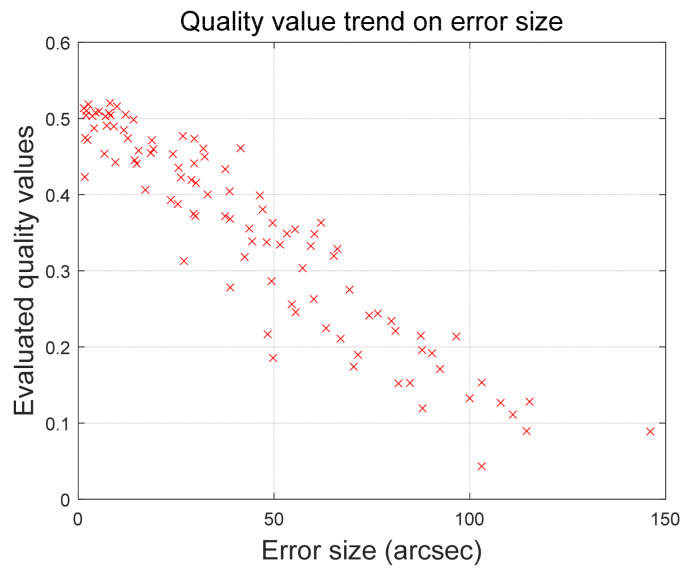
Quality value trend on error size.

The outcomes across various scenarios indicate that incorrect stars exhibit quality values comparable to those of normal stars. However, this resemblance can be attributed to the assumption that errors assigned to incorrect stars follow a random Gaussian distribution. Within the simulation process, instances should arise where the error is approximately 10 arc-seconds. In such cases, the algorithm naturally calculates a quality value similar to that of stars in standard situations. To effectively illustrate whether the proposed work accurately reflects the magnitude of errors, the results of quality value calculations are verified based on the error size. Figure 6 presents the trend of quality values in relation to the error size in Scenario 1.

As depicted in Figure 6, the quality value exhibits a clear linear decrease with increasing error. An additional noteworthy point from the results is that stars with the same error size do not consistently share the same quality value. The proposed methodology utilizes changes in singular values associated with errors to assess the magnitude of the error. However, not all stars show an identical magnitude of singular value change for a given error size. While the variation in singular values due to errors generally displays a similar level of fluctuation on average, individual stars exhibit some variability. As observed in the figure, there is a subtle variation in the quality value even for the same error size.

**Figure 7:**
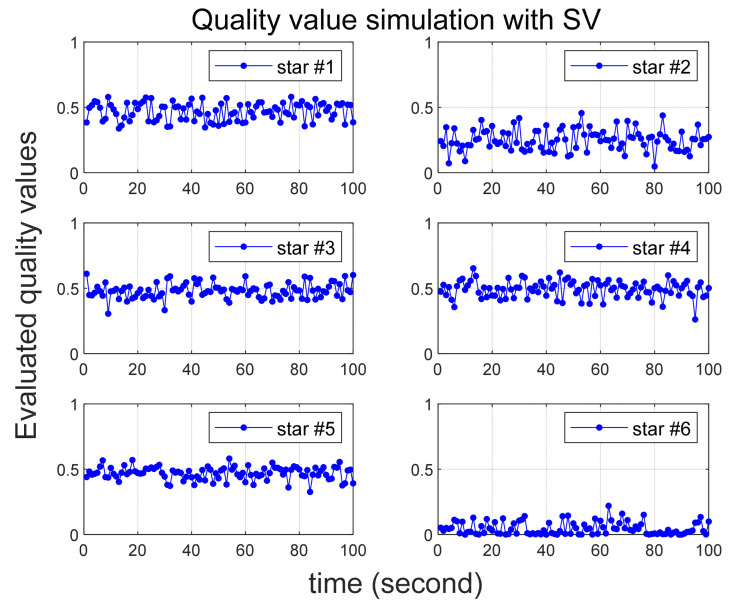
Quality value calculation results with bias type noise.

The current significance of this research lies in its ability to assess and quantify errors that deviate from the normal range. For instance, issues such as the failure of star identification and the deflection of starlight can induce errors in the form of biases. Simulation results in Scenario 1, where the error form is changed from a random Gaussian to a bias, are illustrated in Figure 7. In contrast to prior findings, it is noteworthy that the quality value displays a distinct shifted pattern based on the magnitude of the error. Therefore, from the perspective of fault detection and isolation, the proposed work can significantly contribute to improving attitude determination.

### 4.2. Effects Depending on the Number of Abnormal Stars

The proposed method has proven effective in accurately calculating the quality value through a comparison of singular value variations among recognized stars. However, the key factor closely tied to the performance of the proposed method is the ratio between the number of stars within the normal error range and the number of abnormal stars. This ratio may vary from frame to frame. To validate this, additional simulations are conducted, introducing an increased number of stars with significant errors.

In Scenarios 4 and 5, similar to earlier scenarios, it is assumed that a total of six stars are recognized. However, in each scenario, three and four stars are assumed to have abnormal errors, respectively. Table 4 summarizes the errors associated with abnormal stars, and, for ease of comparison with Figure 7, these errors are presented in terms of bias types.

**Figure 8:**
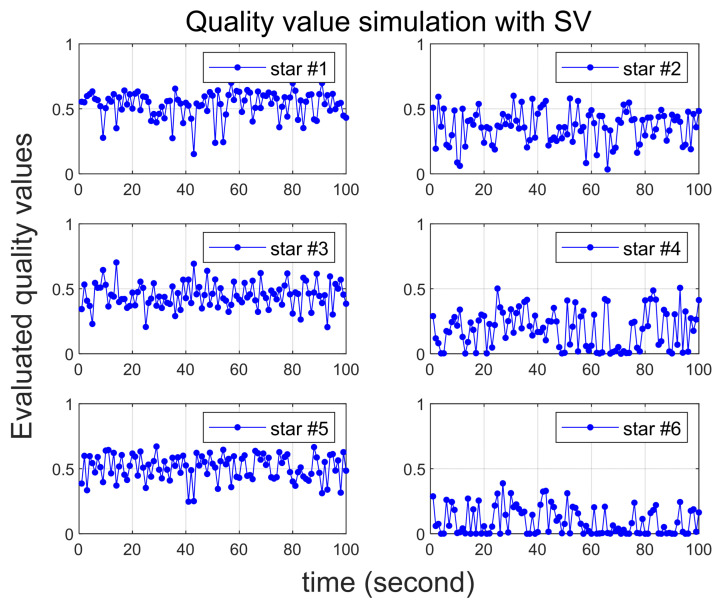
Quality value calculation results under Scenario 4.

In Scenario 4, with the increase in the number of abnormal stars to three, it indicates a situation where only half of the stars in the image frame exhibit normal errors. The results of Scenario 4 are represented in Figure 8. Comparing it to Figure 7, where there are two abnormal stars, it becomes evident that the distinction in quality values is less pronounced. This underscores that the algorithm performs more effectively when the proportion of normal stars within the image frame is higher.

**Figure 9:**
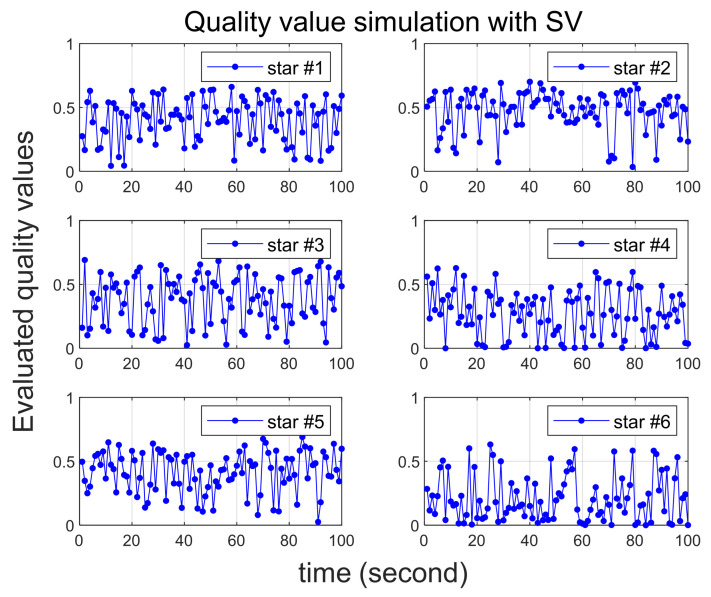
Quality value calculation results under Scenario 5.

Additionally, the outcomes of Scenario 5, where the number of abnormal stars is four, are depicted in Figure 9. In this case, the image frame becomes more dominated by the presence of abnormal stars than normal stars. The 4th and 6th stars, characterized by larger errors, exhibit notably lower quality values than the others. However, distinguishing a meaningful difference in quality values for the 1st and 3rd stars, which have normal errors when compared to stars #2 and #5,^,^ proves challenging.

These outcomes arise from the methodology of computing singular values, which involves grouping three stars rather than assessing individual stars. The proposed approach determines the quality value for a specific star by averaging the assessed quality values of all subgroups containing that star of interest. Consequently, in scenarios where the number of abnormal stars dominates within an image frame, identifying the specific star contributing to the singular value error within that group becomes difficult. This underscores a limitation inherent in the approach utilizing singular values as a reference.

### 4.3. Attitude Determination

**Figure 10:**
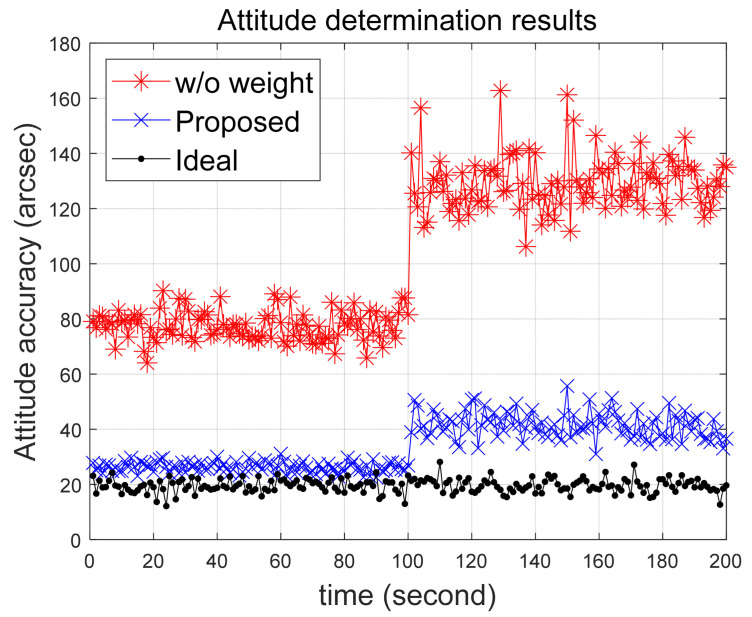
Comparing results of the attitude determination accuracy under Scenario 3.

In this section, the difference in attitude determination accuracy will be compared when the calculated quality value is applied as a weighting factor for each observation versus when it is not applied. For comparison, Scenario 3 previously used to examine the quality value is employed. Additionally, in contrast to the scenarios, an ideal case is introduced where all stars maintain a normal condition, making comparison more straightforward. Attitude determination utilizes the QUEST algorithm as described in the preceding section, with the outcomes presented in Figure 10.

The results indicate that reflecting the error level is effective in enhancing positional accuracy. A crucial aspect of the proposed work is that even in scenarios with incorrect stars, it helps approach the positional accuracy of the ideal case. The process of attitude determination unfolds in three key stages: Sensor Measurement (Vector Observation), Observation Error Level Assessment, Attitude Determination. The primary goal of the presented work is to enhance attitude determination accuracy. Specifically, the focus is on evaluating the quality value of vector observations used in attitude determination. This aligns more closely with the preliminary task of assessing observation error levels rather than committing to a specific attitude determination method.

Various methods are employed for attitude determination, encompassing static approaches like the least square method and QUEST, as well as dynamic methods like the the Extended Kalman Filter (EKF) that integrates gyro data. The pre-evaluated error level can find application in the cost function of the QUEST algorithm or serve as a weighting factor in the EKF update process. Consequently, the presented work holds a significant advantage, being universally applicable and capable of enhancing performance, irrespective of the attitude determination method employed.

**Figure 11:**
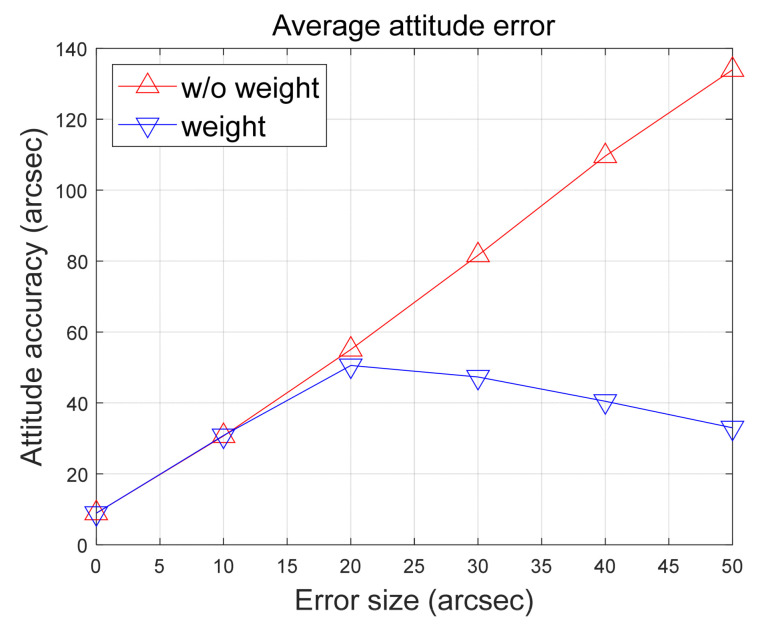
Comparing results of the attitude determination accuracy.

Additional simulation is carried out to observe the trends in algorithm behavior concerning error magnitude. Assuming errors exist in two of the six stars, mirroring Scenario 1, a total of 100 Monte Carlo simulations are executed for each error size. The error magnitude was progressively increased, and the average results were analyzed. Figure 11 exclusively illustrates results up to 50 arc-seconds, offering a detailed analysis of the trend in the algorithm’s accuracy improvement with respect to error size. The noticeable divergence in attitude determination accuracy becomes evident when the error size exceeds 10 arc-seconds.

The observed trend can be directly attributed to the value of $R_{sv}$ used in the quality value calculation. This particular parameter carries significant weight within the algorithm, representing the error of the star sensor under normal conditions. In this study, it is assumed to be 10 arc-seconds. This assumption guides the proposed algorithm to assign a lower weighting factor to star observations with errors larger than those typically encountered.

Ensuring the accuracy of the algorithm requires prior knowledge of errors in the sensor’s typical operating conditions. However, changes in sensor performance can manifest during mission operations. Short-term fluctuations may arise from temperature variations in the space environment, while long-term degradation can occur as hardware approaches the end of its life. Therefore, instead of keeping the value applied to $R_{sv}$ constant, adapting it based on the situation is deemed to significantly enhance algorithm performance.

**Figure 12:**
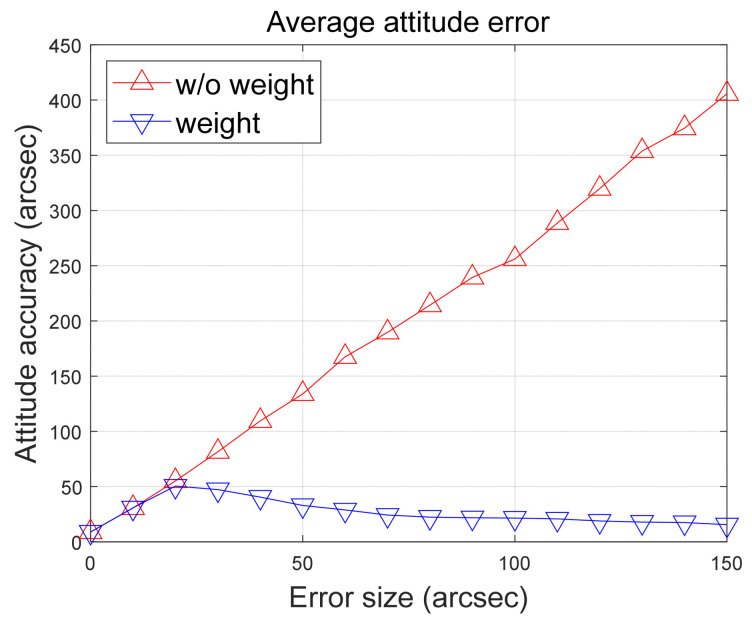
Comparing results of the attitude determination accuracy up to 150 arc-seconds.

In the final analysis, errors were increased up to 150 arc-seconds, and the results are presented in Figure 12. The results demonstrate that when the proposed algorithm is applied as the weighting factor for observations, more precise attitude determination can be achieved. A noteworthy point is that as the error present in an observation increases, the confidence level of that observation decreases, making it a negligible factor in the attitude determination. As a result, utilizing only observations that have higher quality leads to significantly improved attitude accuracy. While the performance improvement is not substantial under normal circumstances, the results reveal significant enhancements as the error value increases. However, the normal sensor behavior cannot always be guaranteed during operations; continuous errors or sudden anomalies like image spikes may occur. In such situations, it is believed that the proposed algorithm can play a pivotal role in enhancing the stability required for the spacecraft’s mission.

## 5. Conclusions

In this study, a novel method is proposed to enhance attitude determination accuracy using star trackers. The key innovation involves the utilization of singular values, which remain invariant despite attitude changes, as reference values for assessing the magnitude of errors introduced in observations. The calculated singular value difference is quantified through hypothesis testing and is subsequently incorporated as an observational weight in the attitude determination process to improve attitude accuracy. Simulations confirm that the algorithm correctly evaluates the quality level. A noteworthy result is that errors attributable to sensor failures are rarely used as observations, which can enhance the reliability of attitude determination. Therefore, it is anticipated that the proposed algorithm can be applied to missions requiring high-precision attitude, as well as contribute to the stability of spacecraft systems.

## Figures and Tables

**Table 1:** Variation and standard deviation of singular values.

	1st Singular Value	2nd Singular Value	3rd Singular Value
Magnitude	1.7288	0.0947	0.0291
Variation w/o noise	3.5374 $\times \;10^{-16}$	7.3239 $\times \;10^{-17}$	5.1625 $\times \;10^{-17}$
Variation w/10 arcsec	2.7827 $\times \;10^{-6}$	3.8214 $\times \;10^{-5}$	3.8230 $\times \;10^{-5}$
Standard deviation w/10 arcsec	2.9367 $\times \;10^{-6}$	4.8097 $\times \;10^{-5}$	4.8389 $\times \;10^{-5}$

**Table 2:** Scenarios for quality value verification.

	Perturbed Star	Error Magnitude (Arc-Seconds)	Simulation Time (s)
Scenario 1	Star #2	25	0 to 100
	Star #6	60	0 to 100
Scenario 2	Star #2	35	0 to 100
	Star #6	70	0 to 100
Scenario 3	Star #2	25	0 to 100
	Star #3	35	100 to 200
	Star #4	70	100 to 200
	Star #6	60	0 to 100

**Table 3:** Identity of image stars utilized in simulation.

Star #1	Star #2	Star #3	Star #4	Star #5	Star #6
89,962	91,117	92,175	90,135	91,845	91,726

**Table 4:** Scenarios for verifying the effects based on the number of abnormal stars.

	Perturbed Star	Error Magnitude (Arc-Seconds)	Simulation Time (s)
Scenario 4	Star #2	25	0 to 100
	Star #4	35	0 to 100
	Star #6	60	0 to 100
Scenario 5	Star #2	25	0 to 100
	Star #4	35	0 to 100
	Star #5	25	0 to 100
	Star #6	60	0 to 100

## Data Availability

The original data are not publicly available but can be used for scientific research. Researchers should email the first author if needed.

## References

[1] Kirkpatrick D., Wertz J.R., Larson W.J., Klungle D. (1999). Space Mission Analysis and Design.

[2] Fallon L., Wertz J.R. (1978). Star Sensors Spacecraft Attitude Determination and Control.

[3] Wang X. (2003). Research on Technology of High-Precision Star Sensor with Large Field of View.

[4] Schulz V.H., Marcelino G.M., Seman L.O., Santos Barros J., Kim S., Cho M., Villarrubia González G., Leithardt V.R.Q., Bezerra E.A. (2021). Universal verification platform and star simulator for fast star tracker design. Sensors.

[5] Liebe C.C. (2002). Accuracy performance of star trackers-a tutorial. IEEE Trans. Aerosp. Electron. Syst..

[6] Lappas V.J., Steyn W.H., Underwood C.I. (2002). Attitude control for small satellites using control moment gyros. Acta Astronaut..

[7] Samaan M.A., Pollock T.C., Junkins J.L. (2002). Predictive centroiding for star trackers with the effect of image smear. J. Astronaut. Sci..

[8] Schiattarella V., Spiller D., Curti F. (2020). Star identification robust to angular rates and false objects with rolling shutter compensation. Acta Astronaut..

[9] Liao Y., Liu E., Zhong J., Zhang H. (2014). Processing centroids of smearing star image of star sensor. Math. Probl. Eng..

[10] Curti F., Spiller D., Ansalone L., Becucci S., Procopio D., Boldrini F., Fidanzati P. (2015). Determining high rate angular velocity from star tracker measurements. International Astronautical Congress: Iac Proceedings.

[11] Kazemi L., Enright J. (2017). Enabling technologies for high slew rate star trackers. Proceedings of the 2017 IEEE Aerospace Conference.

[12] Liebe C.C., Gromov K., Meller D.M. (2004). Toward a stellar gyroscope for spacecraft attitude determination. J. Guid. Control. Dyn..

[13] Zhang W., Quan W., Guo L. (2012). Blurred star image processing for star sensors under dynamic conditions. Sensors.

[14] Wan X., Wang G., Wei X., Li J., Zhang G. (2018). Star centroiding based on fast Gaussian fitting for star sensors. Sensors.

[15] Wan X., Wang G., Wei X., Li J., Zhang G. (2021). ODCC: A dynamic star spots extraction method for star sensors. IEEE Trans. Instrum. Meas..

[16] Accardo D., Rufino G. (2002). Brightness-independent start-up routine for star trackers. IEEE Trans. Aerosp. Electron. Syst..

[17] Padgett C., Kreutz-Delgado K., Udomkesmalee S. (1997). Evaluation of star identification techniques. J. Guid. Control Dyn..

[18] Mu Z., Wang J., He X., Wei Z., He J., Zhang L., Lv Y., He D. (2019). Restoration method of a blurred star image for a star sensor under dynamic conditions. Sensors.

[19] Han J., Yang X., Xu T., Fu Z., Chang L., Yang C., Jin G. (2021). An end-to-end identification algorithm for smearing star image. Remote Sens..

[20] Rijlaarsdam D., Yous H., Byrne J., Oddenino D., Furano G., Moloney D. (2020). A survey of lost-in-space star identification algorithms since 2009. Sensors.

[21] Mehta D.S., Chen S., Low K.S. (2018). A robust star identification algorithm with star shortlisting. Adv. Space Res..

[22] Wei X., Wen D., Song Z., Xi J., Zhang W., Liu G., Li Z. (2019). A star identification algorithm based on radial and dynamic cyclic features of star pattern. Adv. Space Res..

[23] Juang J.N., Wang Y.C. (2012). Further studies on singular value method for star pattern recognition and attitude determination. J. Astronaut. Sci..

[24] Yin H., Song X., Yan Y. (2015). Robustness analysis and improvement of singular value decomposition algorithm for autonomous star identification. Proc. Inst. Mech. Eng. Part G J. Aerosp. Eng..

[25] Wu L., Xu Q., Heikkilä J., Zhao Z., Liu L., Niu Y. (2019). A star sensor on-orbit calibration method based on singular value decomposition. Sensors.

[26] Golub G.H., Van Loan C.F. (2013). Matrix Computations.

[27] Andrews H., Patterson C. (1976). Singular value decompositions and digital image processing. IEEE Trans. Acoust. Speech Signal Process..

[28] Markley F.L. (1988). Attitude determination using vector observations and the singular value decomposition. J. Astronaut. Sci..

[29] Horn R.A., Johnson C.R. (2012). Matrix Analysis.

[30] Hashim H.A., Brown L.J., Mcisaac K. (2018). Guaranteed performance of nonlinear attitude filters on the special orthogonal group SO(3). IEEE Access.

[31] Wahba G. (1965). A least square estimate of satellite attitude. SIAM Rev..

[32] Markley F.L. (1992). Attitude determination using vector observations: A fast optimal matrix algorithm. Flight Mechanics (Estimation Theory Symposium.

[33] Davenport P.B. (1968). A Vector Approach to the Algebra of Rotations with Applications.

[34] Perryman M.A., Lindegren L., Kovalevsky J., Hoeg E., Bastian U., Bernacca P.L., Crézé M., Donati F., Grenon M., Grewing M. (1997). The HIPPARCOS catalogue. Astron. Astrophys..

